# Comprehensive investigation of cuproptosis-related genes in clinical features, biological characteristics, and immune microenvironment in B-cell Non-Hodgkin lymphoma

**DOI:** 10.1515/jtim-2025-0025

**Published:** 2025-07-30

**Authors:** Chengcheng Liu, Ruonan Shao, Xiaoqing Li, Yiran Li, Zhi Tian, Fenling Zhou, Lu Chen, Jiajun Liu, Boyang Chang, Wenjian Liu, Hailin Tang

**Affiliations:** Department of Hematology, The Third Affiliated Hospital of Sun Yat-Sen University; Sun Yat-Sen Institute of Hematology, Guangzhou, Guangdong Province, China; State Key Laboratory of Oncology in South China, Guangdong Provincial Clinical Research Center for Cancer, Sun Yat-Sen University Cancer Center, Guangzhou, Guangdong Province, China; Taneja College of Pharmacy, University of South Florida, Tampa, FL, USA; Institute of Hematology, Jinan University, Guangzhou, Guangdong Province, China; Department of Interventional Radiology, The Third Affiliated Hospital of Sun Yat-Sen University, Guangzhou, Guangdong Province, China

**Keywords:** lymphoma, cuproptosis, immune microenvironment, elesclomol, target therapy

## Abstract

**Background and Objectives:**

Despite the discovery of cuproptosis as a new type of cell death, less is known about the role cuproptosis-related genes (CRGs) may play in B-cell Non-Hodgkin Lymphoma (NHL). There remained a lack of knowledge regarding the clinical and biological roles of CRG signatures and the therapeutic value of the potent copper ionophore (elesclomol) in B-cell NHL. In this study, the purpose is to investigate the prognostic value of CRGs and their relationship to the tumor immune microenvironment, as well as the mechanism of cuproptosis in B-cell NHL.

**Methods:**

B-cell NHL patients’ clinical and gene expression data were retrieved from Gene Expression Omnibus (GEO). Our prognostic model was developed using least absolute shrinkage and selection operator (LASSO) regression analysis and univariate Cox analysis. Prediction accuracy of the model was estimated by receiver operating characteristic (ROC) curves. Functional pathway enrichments and immune features were also analyzed. Vitro experiments were conducted to investigate the combination therapy of elesclomol and doxorubicin, and to explore the application value in B-cell NHL.

**Results:**

Seven CRGs were strongly associated with patient survival and 4 genes were identified to construct the prognostic model. ROC curves indicated great predictive sensitivity and specificity of the model in all cohorts. Patients were divided into low-and high-risk groups by median risk score in each cohort and the survival of the low-risk group was significantly superior than that of the high-risk group. Correlations with clinical features showed that higher Risk-Score was significantly associated with advanced Ann Arbor stages, which were further confirmed in two validation cohorts. We also observed a close relationship between functional pathways and immune features with risk scores. Moreover, we combined elesclomol and doxorubicin in our *in vitro* experiments and found synergetic antitumor effects of the two agents, and the underlying mechanism is the overgeneration of intracellular Reactive Oxygen Species (ROS).

**Conclusions:**

We demonstrated the important value of CRG signatures in prognosis of B-cell NHL patients, and that may be a potential antitumor target for B-cell NHL.

## Introduction

Despite advancements in therapeutic methods, the prognosis of Non-Hodgkin Lymphoma (NHL) remains suboptimal due to the presence of refractory and relapsed cases. NHL, a heterogeneous group of cancers, can be categorized into B cell, T cell, or natural killer-cell NHL.^[1, 2, 3]^ NHL is characterized by a more complex nature compared to Hodgkin lymphoma (HL), stemming from differences in cell origins, diverse biological characteristics, and clinical behavior. Patients with NHL exhibit a poorer prognosis, as evidenced by the estimated 68,500 new cases and 37,600 related deaths in China in 2016.^[4]^ In 2024, Hematologic malignancies, including non-Hodgkin lymphoma (NHL), account for approximately 5% of all cancer diagnoses. B-cell NHL is the most common subtype, representing 85%–90% of NHL cases, with diffuse large B-cell lymphoma (DLBCL) and follicular lymphoma (FL) dominating its epidemiology. ^[5,6]^ In the US, the overall 5-year relative survival rate for B-NHL is 74%. Various treatments, including chemotherapy, targeted therapy, stem cell transplantation, and chimeric antigen receptor (CAR) T-cell therapy, have shown promise in improving clinical outcomes and prognosis for B-cell NHL patients.^[4,7, 8, 9]^ Nevertheless, challenges persist, particularly in managing relapsed or refractory cases.^[10,11]^ Prognostic biomarkers are critical for personalizing treatment and addressing the heterogeneity of B-cell NHL. Therefore, further investigations into novel therapeutic agents are required in treatment of B-cell NHL.

Cuproptosis, a recently identified copper-dependent pathway of cell death, underscores the critical role of maintaining appropriate copper levels in sustaining organismal function and homeostasis.^[12,13]^ Dysregulation of copper levels can impair the activity of copper-binding enzymes, leading to cellular damage, while excessive copper accumulation can trigger cell death.^[12,14,15]^ Notably, recent research by Peter *et al*. has demonstrated that the toxicity of copper is attributed to the metal itself rather than copper ionophores.^[16]^ Cuproptosis represents a different form of cell death, distinct from established mechanisms such as apoptosis, ferroptosis, and necrosis. It relies on mitochondrial respiration rather than adenosine triphosphate (ATP) production.^[16,17]^

Recent research indicates that copper plays a significant role in various biological processes in cancer, such as autophagy, lipolysis, and cell growth. This suggests that cuproptosis could be a potential mechanism for cancer treatment. However, further investigation is needed to fully understand the regulatory mechanisms of this newly identified form of cell death. Previous studies have identified novel genes associated with ferroptosis and necroptosis.^[18, 19, 20]^ However, the existing literature on the genes responsible for regulating cuproptosis in cancers is limited, with only a small number of studies addressing this topic. Research on the association between cuproptosis and B-cell NHL is also lacking. Consequently, further investigation into the cuproptosis-related regulatory network is warranted. It is hypothesized that additional novel genes play a role in the cuproptosis process in B-cell NHL. Furthermore, elucidating their involvement in regulating cuproptosis in B-cell NHL is crucial for advancing our understanding of this phenomenon.

The development of copper-regulating genes (CRGs) signatures and the discovery of PDHA1 in cuproptosis regulation have the potential to enhance clinical decision-making for individuals with B-cell NHL. This study analyzed the correlation between CRGs and the prognosis of B-cell NHL using a publicly available database. The objective of this study is to enhance treatment sensitivity and improve B-cell NHL patient survival rates by providing a more accurate prognosis and guiding treatment selection.

## Materials and methods

### Data acquisition

Transcripts and clinical data of six B-cell NHL cohorts, including GSE16131, GSE32018, GSE10846, GSE32918, GSE23501, and GSE4475 were sourced from the Gene Expression Omnibus (GEO, https://www.ncbi.nlm.nih.gov/geo/). B-cell NHL patients with complete clinical data, including age, LDH ratio, IPI score, Ann Arbor stage, ECOG performance status, number of extra-nodal sites, and survival status was included in the study.^[21,22]^ The GSE32018 cohort includes patients with “normal contributors (Normal, 7 samples), nodal marginal zone lymphoma (NMZL, 13 samples), marginal zone lymphoma of mucosa-associated lymphoid tissue (MALT, 15 samples), follicular lymphoma (FL, 23 samples), mantle cell lymphoma (MCL, 24 samples), diffuse large B cell lymphoma (DLBCL, 22 samples).^[23]^ The Robust Multichip Average (RMA) algorithm was employed for background correction and normalization.^[24]^ The CRGs list is based on previous literature.^[16]^ Ultimately, our study incorporated eleven CRGs: *ATP7A, ATP7B, PDHA1, PDHB, DBT, DLD, DLAT, DLST, FDX1, LIAS*, and *LIPT1*.

### Development and validation of a prognostic model based on cuproptosis-related genes

The GSE16131 cohort was chosen as the training set for developing the prognostic model, with candidate prognostic genes significantly linked to B-cell NHL prognosis identified through Kaplan-Meier analysis (*P* < 0.05). Utilizing the LASSO algorithm, commonly employed in medical research, a prognostic signature was constructed.^[25]^ It was refined using 10-fold cross-validation and maximum likelihood estimation to determine the essential penalty parameter λ values using the penalized maximum likelihood estimator. B-cell NHL patients were classified into low-risk or high-risk subgroups based on their IPI scores for further analysis. The predictive performance of the CRG model was assessed using a time-dependent ROC curve. The GSE10846, GSE32918, GSE23501 and GSE4475 cohorts were chosen as the validation sets, and risk scores for participants were calculated using the standardized formula developed in the training set. The study analyzed the correlation between overall survival (OS) and Cuproptosis Score using Kaplan-Meier curves. Subsequently, ROC curves were employed to assess the predictive capability of Cuproptosis Score for 1-, 3-, 5-, and 7-year outcomes. Uni- and multi-variate Cox regression analyses were performed to determine the prognostic independence of the Cuproptosis Score alongside various clinical factors in February 2023.

### Functional enrichment analysis

We used Kyoto Encyclopedia of Genes and Genomes (KEGG) gene sets from the MSigDB database for Gene Set Enrichment Analysis (GSEA).^[26,27]^ Symbol gene sets were used to explore potential biological functions linked to risk scores, with a normalized *P* value < 0.05 deemed statistically significant. Using a single-sample Gene Set Enrichment Analysis (ssGSEA),^[28,29]^ we calculated enrichment scores for 13 immune-related networks. Moreover, we determined the infiltrating score of 22 immune cells, using the ESTIMATE algorithm of the “estimate” package.^[30]^

### Cell viability and cell death assays

Cells were seeded at 8000 cells per well in 96-well plates and treated with Doxorubicin (Selleck) and/or Elesclomol-Cucl2 (Elesclomol, Selleck; Cu2+, Sigma) for 48 hours. Cell viability was evaluated using the Cell Counting Kit-8 following the manufacturer’s guidelines. Cells were treated with CCK8 reagent and incubated at 37°C for 2–3 hours, followed by absorbance measurement at 450 nm using a microplate reader. The Combination Index (CI) of Doxorubicin and Elesclomol (Cu2+) was determined by CompuSyn software program. CI < 1.0 reflects synergism.

Cell apoptosis was measured using the Annexin V-FITC/PI assay kit. Cells were harvested 48 hours after pre-treatment, washed twice in pre-chilled PBS, and resuspended in 500 μL binding buffer. Cells were stained with Annexin V-FITC/PI in the dark for 10–15 minutes per the manufacturer’s instructions and analyzed *via* flow cytometry.

### ROS assay

Intracellular Reactive Oxygen Species (ROS) levels were measured using the DCFH-DA fluorescent probe from the ROS assay kit. After pre-treatment for 48 hours with specific agents, cells were centrifuged at 1000 rpm for 5 minutes, and fluorescent probes were added. The results were quantified using flow cytometry.

### Immunohistochemistry staining

After epitope retrieval, H^2^O^2^ treatment, and blocking of non-specific antigens, tissue sections were deparaffinized, dehydrated, and incubated overnight at 4°C with polyclonal rabbit anti-human PDHA1 antibodies (1:50, Prointech). The secondary antibodies were incubated with the sections at room temperature for two hours, and the signal was visualized using an enhanced DAB staining kit.

### Western blot

We lysed tumor and normal tissues in RIPA buffer with protease and phosphatase inhibitors, then denatured them at 100°C for 15 minutes. The protein samples were separated by 10% SDS-PAGE and transferred to PVDF membranes. The PVDF membranes were blocked with a 5% skim milk powder solution for 1 hour and then incubated overnight with primary antibodies: anti-β-catenin, anti-c-Myc, anti-Casp3, anti-C-Casp3 and anti-GAPDH (1∶5000, Prointech). This was followed by incubation with secondary antibodies (1∶2000, Prointech) for 2 hours at room temperature, observed with the ECL kit chemiluminescence reagent. The Chemidoc system (Bio-Rad, Hercules, CA, USA) was used to detect protein band signals.

### Statistical analysis

The Wilcox test or Student’s *t* test was used for statistical comparison between the two groups. Spearman correlation analysis was utilized to assess the relationship between the expression levels of riskScore and checkpoint-related genes. The Kaplan-Meier survival analysis of the gene signature from the GEO dataset was based on log-rank test comparisons among various groups. The predictive accuracy of genes for overall survival (OS) was assessed using ROC curves generated by the “timeROC” package. Cox regression analyses were conducted to determine the appropriate terms for constructing the nomogram. Statistical analyses were conducted using R software, GraphPad Prism, and SPSS. A *P*-value less than 0.05 was deemed statistically significant.

## Results

### Development and validation of a prognostic CRGs model

**Figure 1:**
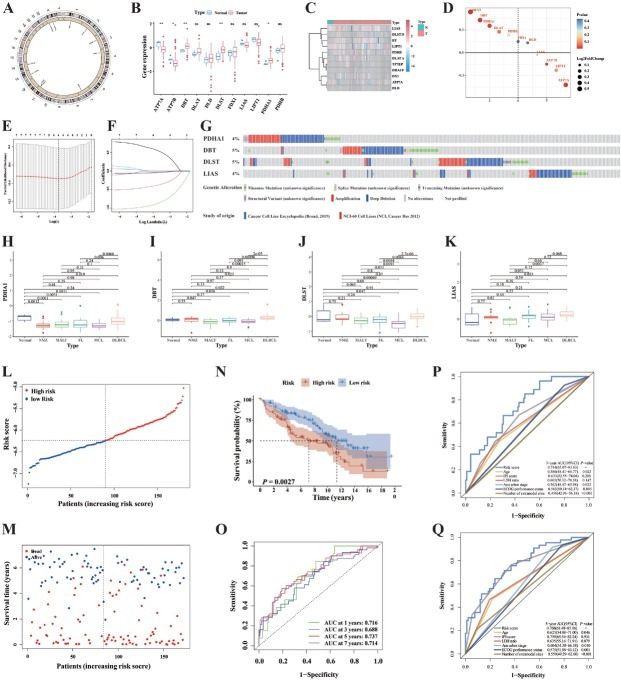
Construction of a prognostic CRG model in the GSE16131 cohort. A. The circos plot illustrating the chromosomal positions of 11 CRGs. B. Expression signatures of 11 CRGs in the GSE32018 cohort (^*^P < 0.05; ^**^P < 0.01). C. Heatmap of 11 GRGs in GSE32018 cohort. D. Gene ranking dotplot of 11 GRGs in GSE32018 cohort. E. 10-fold cross-validation for variable selection in LASSO regression using minimum criteria in the GSE16131 cohort. F. LASSO coefficients of CRGs. Each curve represents a gene. G. Genetic alterations of the four model-linked CRGs in CCLE were obtained from the cBioPortal for Cancer Genomics (http://www.cbioportal.org/). H-K. Expression signatures of 4 model-linked CRGs in the GSE32018 cohort. L. Risk score analysis of 4-CRG profile in the GSE16131 cohort. M. Survival outcome analysis of 4-GRG profile in the GSE16131 cohort. N. Kaplan-Meier curve of 4-CRG profile in the GSE16131 cohort. O. Time-dependent ROC analysis for 1-, 3-, 5-, and 7-year overall survival (OS) of the 4-CRG profile in the GSE16131 cohort. P-Q. Time-dependent ROC analysis for 3- and 5-year OS of the 4-CRG profile was compared to Age, IPI score, LDH ratio, Ann Arbor stage, ECOG performance status, and Number of extra-nodal sites in the GSE16131 cohort. Data were analyzed by Wilcox. Test in Figure 1B, 1D, 1H-1K, log-rank test in Figure 1N, DeLong test in Figure 1P-1Q. NMZL: nodal marginal zone lymphoma; MALT: marginal zone lymphoma of mucosa-associated lymphoid tissue; FL: follicular lymphoma; MCL: mantle cell lymphoma; DLBCL: diffuse large B cell lymphoma.

Supplementary Figure S1 provides an overview of the research design. We identified eleven major CRGs from previously reported noteworthy findings.^[23,24]^ The circos plot illustrates the chromosomal positions of eleven CRGs (Figure1A). The expression patterns of these eleven genes were analyzed using GEO datasets to determine their role in B-cell NHL. Correlation and prognosis analyses indicate that four cuproptosis-related genes are positively correlated and possess prognostic significance in B-cell NHL. Expression signatures of eleven CRGs in the GSE32018 cohort showed that the most strongly associated variables were *ATP7A, ATP7B, DBT, DLST* and *PDHA1* (Figure 1B-1D). Molecular interaction and pathway enrichment analyses reveal that CRGs are associated with oxidative stress, lipid peroxidation, and energy metabolism. Our findings indicate that CRGs could play a significant role in the pathology of B-cell NHL. Lasso regression analysis was subsequently employed to select the four optimal prognosis-related genes and construct the prognostic model (Supplementary Figure S2, Figure 1E and 1F). We analyzed the mutated variants of the four CRGs in the Cancer Cell Line Encyclopedia database *via* cBioPortal for Cancer Genomics. Among the four analyzed CRGs, most mutations were gene deep deletion and amplification (Figure 1G), and the genes *PDHA1, DBT, DLST* and *LIAS* were commonly mutated. Expression signatures of four model-linked CRGs in the GSE32018 cohort (Figure 1H-1K). We subsequently assessed the predictability of the CRG signature in the GSE16131 cohort. These patients were first classified into a HR or LR sub-cohort, depending on median risk score of training cohort. The HR sub-cohort in the GSE16131 cohort experienced higher early mortality compared to the LR sub-cohort. Conversely, the LR sub-cohort demonstrated a favorable prognosis (*P* < 0.001) (Figure 1L and 1M). Kaplan–Meier analysis indicated that low-risk patients had significantly better OS compared to high-risk patients (Figure 1N). We assessed the prognostic model’s efficacy using time-dependent ROC analysis. The area under the ROC curve (AUC) for 1-, 3-, 5-, and 7- year OS was 0.716, 0.688, 0.737, and 0.714 respectively (Figure 1O). We compared various clinical prognostic models to assess the efficacy of the CRG model. The analyses demonstrated that our model outperformed other models in prognosis prediction (Figure 1P and 1Q).

### Identification of the cuproptosis-related genes risk model as an independent prognostic predicting factor for overall survival

**Figure 2:**
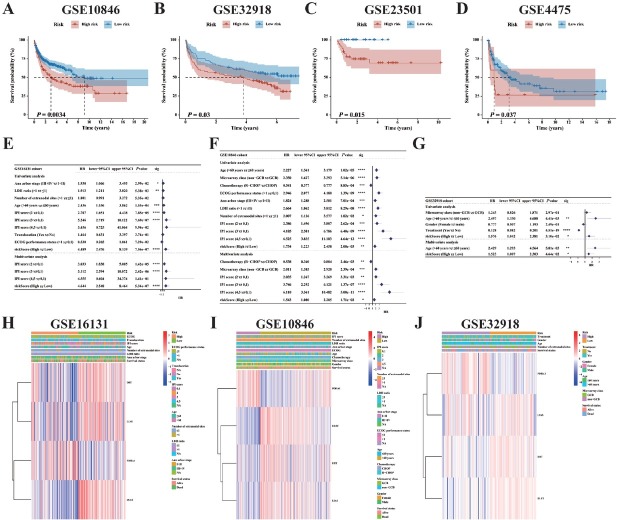
Evaluation and validation of the utility of the prognostic CRG model. A-D. Kaplan-Meier curves of 4-CRG profile in the GSE10846, GSE32918, GSE23501 and GSE4475 cohorts. E-G. Forest plots of the univariate and multivariate Cox analyses in the GSE16131, GSE10846, and GSE32918 cohorts. H-J. Heatmap displaying the 4-CRG profile alongside clinical features across two subgroups in the GSE16131, GSE10846, and GSE32918 cohorts (^*^P < 0.05; ^**^P < 0.01; ^***^P < 0.001; ^***^P < 0.0001). Data were analyzed by log-rank test in Figure 2A-2D.

Significant survival differences between low-risk and high-risk groups were observed in four validation cohorts (*P* < 0.05, Figure 2A-2D). Univariate and multivariate Cox analyses for Age, IPI score, and other prognostic values were conducted across the GSE16131, GSE10846, and GSE32918 cohorts. The univariate Cox analysis identified Age, IPI score, and risk score as independent prognostic indicators for OS across all three cohorts (Figure 2E-2G). We examined the clinical features of two subgroups and identified distinct clinical characteristics between the low-risk and high-risk groups. Patients with early-stage B-cell NHL were predominantly in the low-risk group, whereas those with advanced stages were more frequently classified into the high-risk group. This could account for the improved survival status observed in the low-risk group. The results were consistent across all three cohorts (Figure 2H-2J).

### Establishment and validation of the predictive nomogram

**Figure 3:**
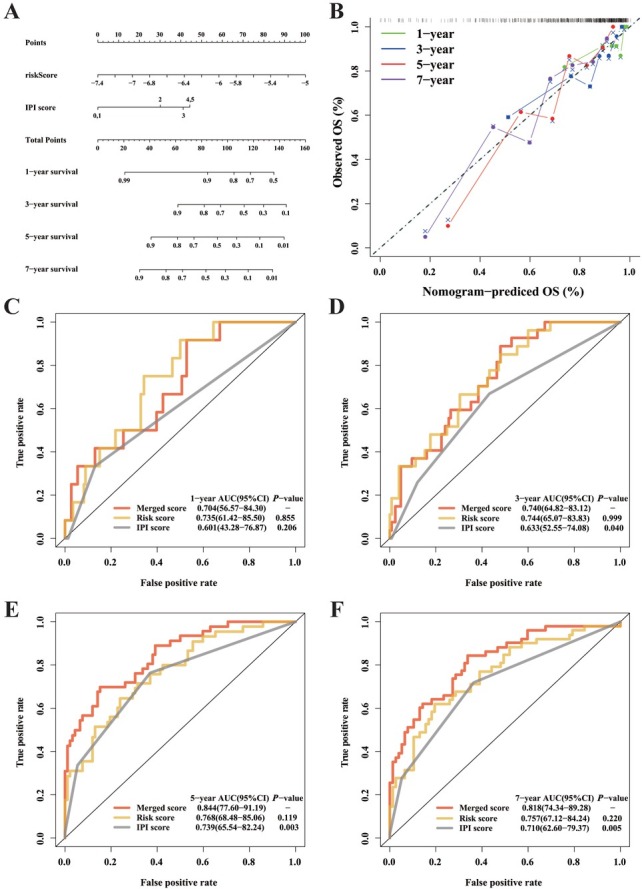
Development and validation of a nomogram for predicting overall survival in lymphoma patients within the GSE16131 cohort. A. Nomogram was built based on risk score and IPI score in the GSE16131 cohort. B. Calibration plot for the nomogram. C-F. Time-dependent ROC curves of nomograms were compared based on 1-, 3-, 5-, and 7-year OS of the GSE16131 cohort. Data were analyzed by DeLong test in Figure 3C-3F.

We performed univariable and multivariable Cox analyses on baseline clinical characteristics and risk scores to identify independent prognostic factors in the training cohort. Univariable analysis indicated that both IPI score and risk score were linked to poorer OS. In the multivariable analysis, both IPI score and risk score remained independent predictors of OS (Figure 3A) and were utilized to create the nomogram depicted in Figure 3B. Calibration plots demonstrated strong alignment between the nomogram-predicted and actual probabilities of 1-, 3-, 5-, and 7-year OS in the GSE16131 cohort (Figure 3B). The merged score’s 1-, 3-, 5-, and 7-year OS AUC significantly surpassed that of the IPI score, indicating that the nomogram improves OS prediction compared to standard prognostic factors (Figure 3C-3F, Supplementary Figure S3).

### Functional analysis in 3 cohorts

**Figure 4:**
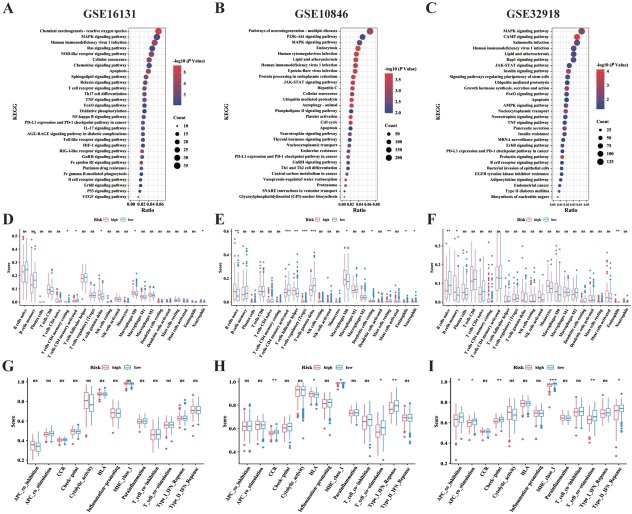
Functional enrichment analysis and tumor immune micro-environment landscape between high- and low-risk subgroups. A-C. Top 30 representative KEGG pathways in high-risk group in the GSE16131, GSE10846, and GSE32918 cohorts. D-F. The enrichment scores of 22 immune cells of two subgroups in the GSE16131, GSE10846, and GSE32918 cohorts. G-I. Enrichment scores for 13 immune-related functions across two subgroups in the GSE16131, GSE10846, and GSE32918 cohorts (^*^P < 0.05; ^**^P < 0.01; ^***^P < 0.001). Data were analyzed by Benjamini-Hochberg test in Figure 4A-4C, Wilcox test in Figure 4D-4I.

We conducted GSEA to determine the biological functions and signaling pathways associated with the risk score. Multiple biological processes and pathways related to cuproptosis, including MAPK, CAMP, oxidative phosphorylation,^[31]^ and RAS signaling pathways, were enriched across three cohorts (Figure 4A, 4B and 4C). Immune-related cellular functions and molecular signaling, including negative regulation of macrophage apoptotic process, macrophage fusion, B cell receptor signaling pathway, T cell receptor signaling pathway, and primary immunodeficiency, were identified in the GSE16131, GSE10846, and GSE32918 cohorts. ESTIMATE algorithm and ssGSEA were performed to explore the relationship with immune cells (Figure 4D, 4E and 4F) and immune function (Figure 4G, 4H and 4I) enrichment with risk score. In the training cohort, the high-risk group exhibited significantly different enrichment scores for T cells gamma delta, macrophages M0, resting mast cells, and activated CD4 memory T cells compared to the low-risk group.^[32,33]^ In the GSE10846 validation cohort, the low-risk group exhibits higher enrichment scores for T cells gamma delta, T cells CD4 memory activated, and mast cells compared to the high-risk group.^[34]^ Significant differences in CCR, Checkpoint, T cell co-stimulation, and Type I-IFN Response scores were observed between the low-risk and high-risk groups in both cohorts. No difference in immune-related functions was observed between the two groups in the GSE16131 cohort.

### Correlation analysis of immune checkpoints in B-cell NHL microenvironment

**Figure 5:**
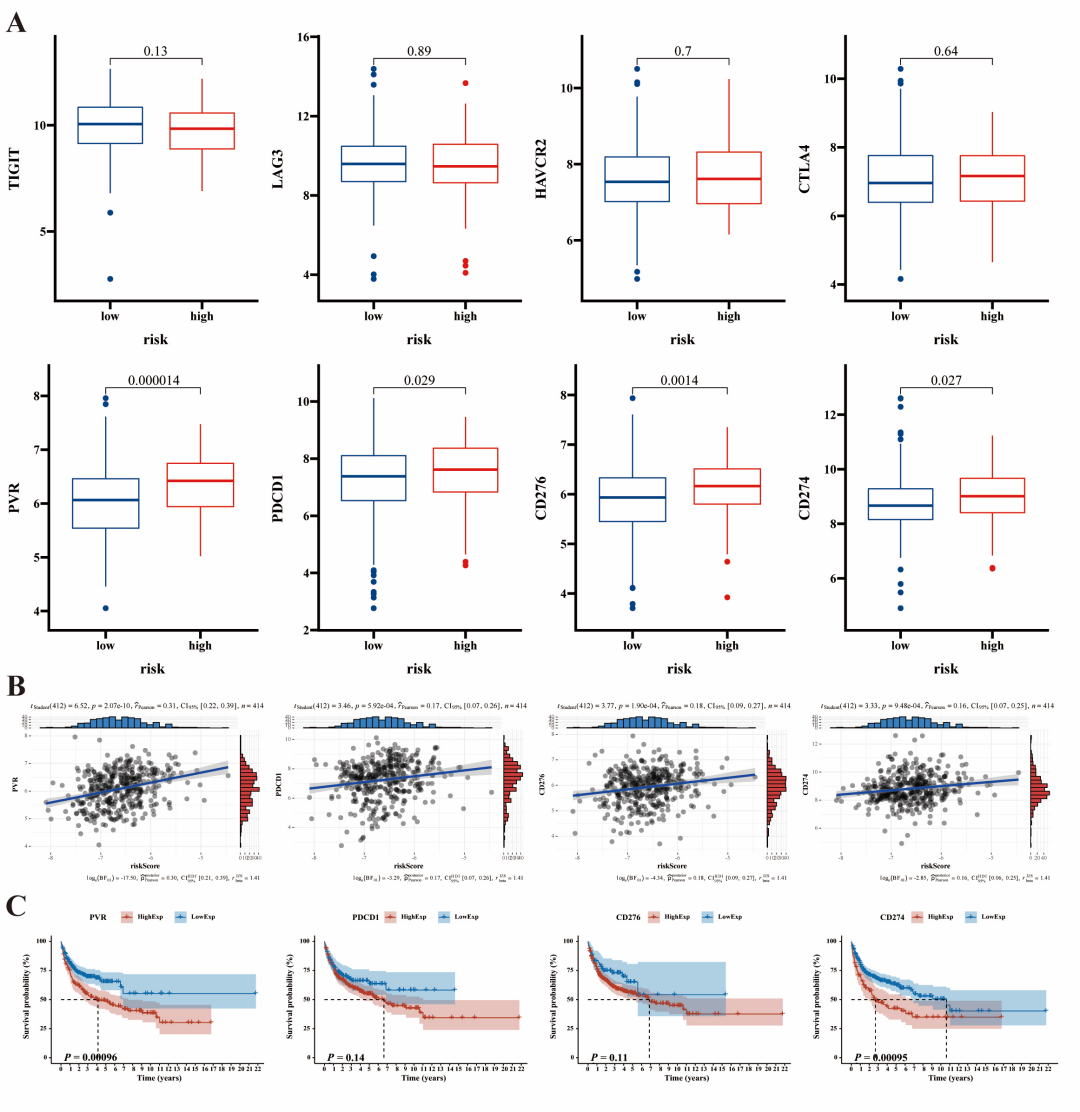
Investigations of immune checkpoints in the GSE10846 cohort. A. Expression levels of 8 immune checkpoints including TIGIT, LAG3, HAVCR2, CTLA4, PVR, PDCD1, CD276 and CD274 in the GSE10846 cohort. B. The correlation between risk score and 4 immune checkpoints including PVR, PDCD1, CD276 and CD274 in the GSE10846 cohort. C. Kaplan-Meier analyses of OS based on expression levels of PVR, PDCD1, CD276 and CD274 in the GSE10846 cohort. Data were analyzed by Wilcox. Test in Figure 5A, two-sided Spearman test in Figure 5B, log-rank test in Figure 5C.

The expression levels of PVR, PDCD1, CD276 and CD274 were higher in high-risk subgroup in the GSE10846 cohort (Figure 5A). Spearman correlation analysis confirmed a positive correlation between PVR, PDCD1, CD276, CD274, and risk score (Figure 5B). The Kaplan–Meier analysis of the GSE10846 cohort revealed a correlation between PVR, CD274 overexpression, and poor OS (Figure 5C). These results provide evidence that B-cell NHL patients with higher CRG riskScore might obtain enhanced response to therapies targeting the checkpoints above.

### Expression of PDHA1 in B-cell NHL

**Figure 6:**
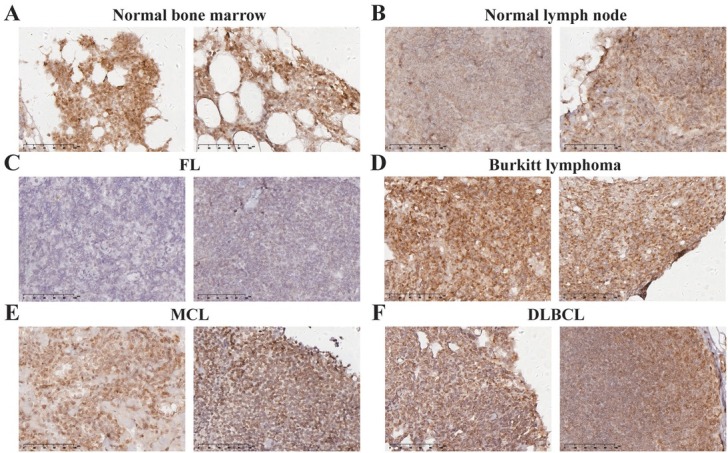
Expression patterns of PDHA1 in normal bone marrow and lymph node tissues (A-B). Expression patterns of PDHA1 in FL, Burkitt lymphoma, MCL and DLBCL (C-F). FL: follicular lymphoma; MCL: mantle cell lymphoma; DLBCL: diffuse large B-cell lymphoma.

Immunohistochemistry analysis revealed that PDHA1 was overexpressed in DLBL, MCL, and Burkitt lymphoma tissues compared to normal node tissues (Figure 6A-6F). These findings confirm PDHA1’s potential oncogenic role and its significance in cuproptosis within DLBCL, MCL, and Burkitt lymphoma. PDHA1 is a potential antitumor target for treating DLBCL, MCL, and Burkitt lymphoma. PDHA1 activation may enhance oxidative phosphorylation, leading to increased oxygen consumption and reduced lactate production in lymphoma cells.

### Combination of elesclomol and doxorubicin synergistically inhibited cell proliferation, induced cell apoptosis, and stimulated ROS accumulation

**Figure 7:**
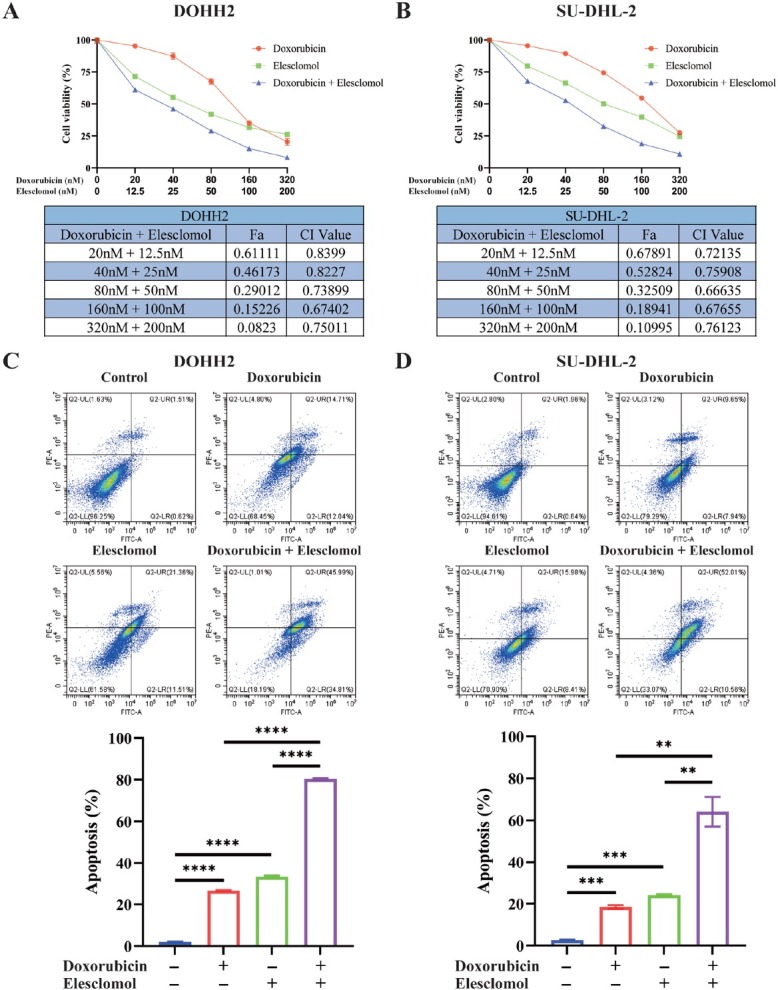
Doxorubicin and elesclomol synergistically enhance cell proliferation inhibition and apoptosis induction. A-B. DOHH2 and SU-DHL-2 cells were treated with varying doses of doxorubicin and elesclomol-Cu, both individually and in combination, for 48 hours. Cell viability was subsequently assessed using CCK8, with a CI value of less than 1 indicating synergy. C-D. Flow cytometry using Annexin V-FITC/PI double staining assessed apoptosis in DOHH2 and SU-DHL-2 cells following 48-hour treatments with DMSO, doxorubicin (40 nM), and/or elesclomol (12 nM)-Cu (CuCl, 0.5 μmol/L). Results are expressed as mean ± _2_SD. Experiments were conducted a minimum of three times. ^**^P < 0.01; ^***^P < 0.001; ^****^P < 0.0001. Data were analyzed by one-way ANOVA adjusted for multiple comparisons.

Elesclomol is a well-studied cuproptosis inducer that works by increasing intracellular copper levels. Co-treatment of elesclomol-Cu and doxorubicin synergized in inhibition of cell proliferation and induction in cell apoptosis. The combination of the two drugs synergistically inhibited cell viability at CI values < 1 (Figure 7A and 7B). Flow cytometry revealed a higher rate of cell apoptosis with co-treatment compared to monotherapies (Figure 7C and 7D).

### ROS production is the key cytotoxic mechanism of cuproptosis

**Figure 8:**
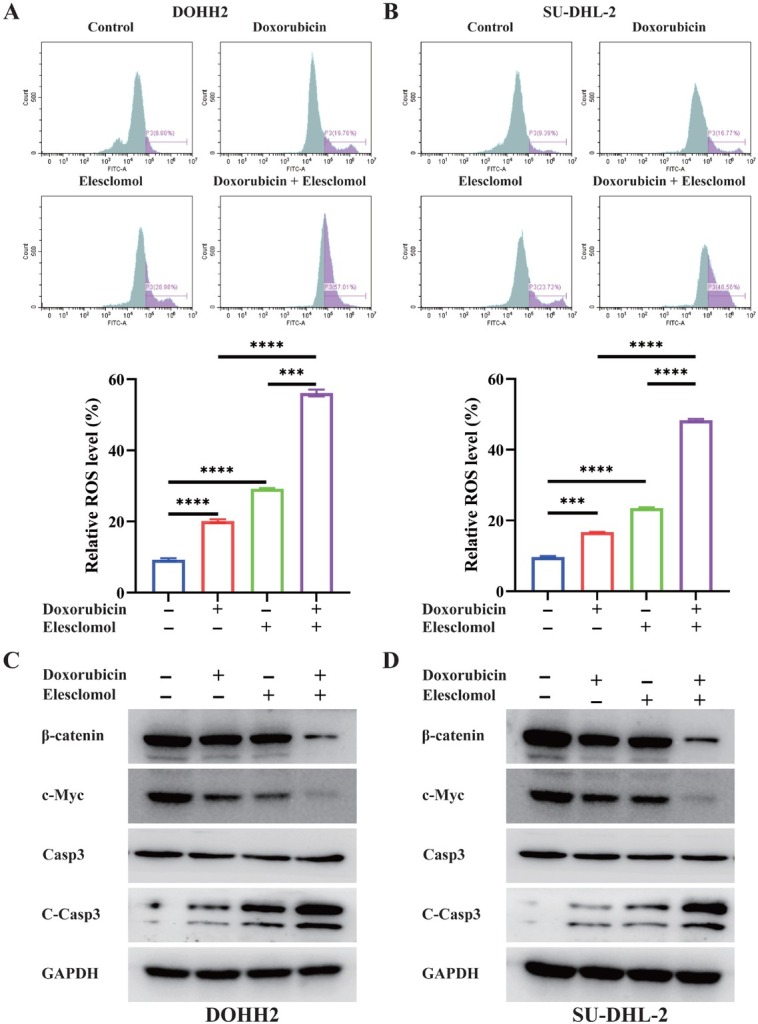
Intracellular ROS production was assessed *via* flow cytometry after 24-hour treatments with DMSO, doxorubicin (40 nM), and/or elesclomol (12 nM)-Cu (CuCl_2_, 0.5 μM) (A-B). Protein levels of β-catenin, c-Myc, Caspase-3, and Cleaved Caspase-3 were analyzed by western blot, using GAPDH as a loading control, following treatments with DMSO, doxorubicin, and/or elesclomol-Cu (C-D). ^***^P < 0.001; ^****^P < 0.0001. Data were analyzed by one-way ANOVA adjusted for multiple comparisons.

As demonstrated by flow cytometry (Figure 8A and 8B). Elesclomol-Cu or doxorubicin may induce intracellular ROS production in lymphoma cell lines (Figure 8A and 8B). The combination of the two drugs significantly increased ROS accumulation. We analyzed the protein levels of various cuproptosis-related markers to explore the molecular mechanisms linking the drug combination, ROS production, and cuproptosis. Treatment with elesclomol-Cu and doxorubicin significantly decreased β-catenin and c-Myc protein levels, while increasing C-Casp3 protein levels in B-cell NHL cell lines (Figure 8C and 8D). The mechanistic specificity of copper-induced cell death remains debated, despite initial studies indicating that cuproptosis operates independently of ROS. PDHA1 may be involved in regulating c-Myc and C-Casp3 through the modulation of Wnt/β-catenin signaling.

## Discussion

B-cell NHL is a common hematological malignancy. Survival outcomes of B-cell NHL patients are influenced by factors such as physical condition, tumor biological behavior, gene expression profiles, and cytogenetic abnormalities.^[35,36]^ The widely recognized IPI score prognostic model is closely associated with patient survival. However, only a few studies have evaluated the prognostic impact of gene expression profiles in B-cell NHL.

Cuproptosis is a unique form of cell death linked to an ancient mechanism.^[16,37]^ However, due to the influence of various factors, the correlation between cuproptosis regulatory factor and molecular model, tumor subtype and prognostic value is not clear, also the role of cuproptosis regulatory factor in the immune microenvironment is not clear.^[38]^ In-depth research on these issues is essential to support the development of targeted drugs and meet specific treatment requirements. Identifying the molecular pattern linked to cuproptosis will enhance our comprehension of cuproptosis and its characteristics in B-cell NHL. Cuproptosis-associated gene patterns can serve as prognostic markers and indicators of therapeutic responses in cancer.

Due to their need for elevated levels of metals like iron and copper to sustain rapid metabolism, cancer cells may be targeted by cuproptosis, which could function as an immunosuppressor and play a role in combination therapy. However, limited research has explored the roles of copper metabolism and homeostasis in cancer cells.^[39, 40, 41]^ Excess copper was thought to kill cells by catalyzing the production of toxic reactive oxygen species. No research has documented the role of cuproptosis in tumor immunity within B-cell NHL.

This study found that most cuproptosis-associated genes were significantly differentially expressed in B-cell NHL samples, with some closely linked to B-cell NHL prognosis. We conducted regression analysis to classify B-cell NHL into two subgroups based on CRG expression. Significant differences were observed between the two subtypes in terms of immune cells, immune checkpoint status, prognosis, and treatment response. PDHA1 was identified as a robust, independent, and consistent clinical predictor of OS in B-cell NHL patients. PDHA1 is primarily involved in oxidative processes and stress responses to copper/metal ions. Multi-dimensional analyses evaluated the relationship between TME, immunotherapy, and therapeutic sensitivity of fourteen CRGs in B-cell NHL. The findings suggest that PDHA1 may affect B-cell NHL prognosis by influencing the immune microenvironment and therapy sensitivity. To our knowledge, this is the first study to reveal the involvement and potential regulatory mechanisms of the cuproptosis-related gene PDHA1 in B-cell NHL. This study developed a prognostic model incorporating CRGs associated with B-cell NHL survival, demonstrating greater accuracy in survival prediction compared to IPI. The gene expression profile of B-cell NHL in this study offers a foundation and promising targets for novel drug exploration.

Previous studies have confirmed the involvement of ferroptosis, necroptosis, and pyroptosis in immunooncology.^[42, 43, 44]^ We propose a significant association between cuproptosis and tumor immunity. CD8+ T cells have been shown to induce ferroptosis in tumor cells, while natural killer (NK) cells and cytotoxic T lymphocytes inhibit tumor cells through pyroptosis.^[45, 46, 47]^ We utilized CIBERSORT to determine the proportions of various tumor-infiltrating immune cells in B-cell NHL, aiming to elucidate the potential immune landscape of cuproptosis in B-cell NHL. Patients in the high-risk group exhibited downregulated levels of various functional immune cells, including B cells, plasma cells, CD4 memory T cells, M2 macrophages, eosinophils, and immature immune cells like M0 macrophages. The levels of these immune cells were significantly associated with the progression of B-cell NHL patients.^[48,49]^ Immunological evasion in high-risk B-cell NHL patients may result in suboptimal prognoses. Alternatively, these functional immune cells could undergo cuproptosis. Similar findings were observed in immunological states analysis. We assessed the enrichment scores for 22 immune cell types and 13 immune-related functions across the two risk groups. B cells, DCs, iDCs, mast cells, Th2 cells, Treg cells, and NK cells were downregulated in the high-risk group. Immune processes were inhibited in the high-risk group, with survival analysis indicating restrained immune cell proliferation and functions, resulting in poorer OS rates. We observed significant immune inhibition in the high-risk group and considered whether cuproptosis inducer therapy could benefit these patients. The functional pathways are closely related to cuproptosis, such as oxidative phosphorylation, may be associated with the immune microenvironment in B-cell Non-Hodgkin Lymphoma. For example, changes in these pathways can affect the recruitment and activation of immune cells. The risk scores we calculated also can be used as a potential biomarker to predict patient prognosis and response to treatment. However, further research is required to understand the roles of these markers in B-cell NHL progression. Furthermore, there is a lack of sufficient data to investigate their roles in cuproptosis. Our comprehensive exploration of these cuproptosis markers indicates their significant impact on the development and progression of B-cell NHL. Cuproptosis may be linked to the immune environment of B-cell NHL.^[50,51]^

The detailed mechanisms underlying copper-related toxicity in organisms remain inadequately understood. Previous research has proposed various perspectives, including the induction of apoptosis, caspase-independent cell death, oxidative stress, and extracellular ATP release.^[52, 53, 54, 55, 56, 57, 58]^ In the study conducted by Tsvetkov *et al*., the authors posited that cells engaged in aerobic respiration (referred to as TCA-cycle active cells) exhibited elevated levels of lipoylated TCA enzymes, which were capable of directly binding copper, thereby leading to the lipoylation of proteins and the loss of Fe-S cluster–containing proteins, finally leading to acute proteotoxic stress and cell death.^[16,59]^ This study represents the first to establish the precise concept that copper-induced cell death is mediated through mitochondrial function. Additionally, it provides comprehensive insights into the mechanisms and implications of copper-induced cell death, warranting further investigation in future research.

Elesclomol, a cuproptosis inducer, binds to VDAC2, maintaining its opening and altering mitochondrial membrane permeability, which increases mitochondrial metabolism and ROS production.^[60, 61, 62]^ Doxorubicin is a traditional chemotherapy agent used to treat B-cell NHL. Combining effective small-molecule drugs with low-dose doxorubicin enhances its cytotoxicity against tumor cells, achieving desirable antitumor effects while reducing doxorubicin’s toxic side effects.^[63, 64, 65, 66]^ Research indicates that doxorubicin, either alone or in combination with other agents, induces ROS production.^[67,68]^ Our *in vitro* experiments have proved the synergistic effect of elesclomol and doxorubicin against B-cell NHL cell lines. The mechanism involves significantly elevated intracellular ROS levels in the combination group. The potential mechanisms may be that elesclomol can enhance copper-mediated oxidative stress, and doxorubicin is known to generate ROS through its interaction with cellular components. High ROS levels can cause damage to cellular macromolecules such as DNA, proteins, and lipids, ultimately leading to cell death. Furthermore, preliminary studies have explored the molecular mechanisms. Cuproptosis is a novel copper-dependent mechanism of cell death. Evidence indicates that imbalanced copper levels can affect tumor growth and cause various forms of cell death, and copper is closely linked to antitumor immunity and immunotherapy.

Our study still has several limitations. The prognostic model data were retrospectively sourced from public databases and require validation through real-world and prospective studies. Second, relying on a single prognostic gene model for survival prediction has inherent limitations, necessitating the inclusion of genes associated with other characteristics to enhance the model. Next, further mechanisms such as changes in Wnt/β-catenin signaling pathway and oxidative phosphorylation level caused by elesclomol still need to be studied in the future. Finally, *in vitro* experiment results must be validated by *in vivo* experiments. We have outlined our follow-up work plans. In the near future, we plan to conduct *in vivo* experiments using animal models of B-cell Non-Hodgkin Lymphoma to validate the *in vitro* findings. We also aim to collect more clinical data to further refine the risk score model and test its predictive power in a larger patient cohort. Additionally, we will explore the molecular mechanisms underlying the observed relationships in more detail, for example, by using gene knockdown and overexpression techniques.

To sum up, we developed a prognostic model for B-cell NHL including 4 CRGs, which exhibited great prognostic accuracy in both training and validation cohorts. Moreover, a synergistic effect of the cuproptosis inducer elesclomol and the classical chemotherapeutic agent doxorubicin was illustrated *in vitro*. These findings suggest that cuproptosis could be a valuable metric for survival prediction and an antitumor target in B-cell NHL, aiding in patient diagnosis, prognosis, and clinical trial design. An examination of immune infiltration and cuproptosis was conducted. Furthermore, the study laid a preliminary foundation for researching the mechanisms of cuproptosis.

In conclusion, our study investigated the expression patterns of eleven cuproptosis markers and developed a robust prognostic predictive model using four CRGs. We performed a preliminary analysis on PDHA1’s regulatory role in cuproptosis. The proposed signature in this study shows potential as a clinical biomarker and candidate target for B-cell NHL. It can help predict the immunotherapy sensitivity of B-cell NHL patients in clinical practice. Enhancing doxorubicin sensitivity in B-cell NHL cells with the cuproptosis inducer elesclomol may offer a promising treatment strategy for B-cell NHL. Cuproptosis, a recently identified form of cell death, shows promising potential as a novel cancer treatment. Identifying new cell death types, such as cuproptosis, could provide alternative treatment strategies for overcoming drug resistance in refractory cancer cases, as apoptosis is the primary mechanism mediating chemotherapy resistance.

## Supplementary Information

Supplementary materials are only available at the official site of the journal (www.intern-med.com).

## Supplementary Material

Supplementary Materials
