# Supplementary material for: Comprehensive investigation of cuproptosis-related genes in clinical features, biological characteristics, and immune microenvironment in B-cell Non-Hodgkin lymphoma
Source: J Transl Int Med. 2025 Jul 30;13(5):456–71. doi: 10.1515/jtim-2025-0025 (PMC12569579; doi:10.1515/jtim-2025-0025)
Supplement: Supplementary file 1 — Supplementary Materials [file jtim-2025-0025_sm.pdf]

Supplementary materials

Comprehensive investigation of cuproptosis-related genes in clinical features, biological characteristics, and immune microenvironment in B-cell Non-Hodgkin Lymphoma

Chengcheng Liu[1#], Ruonan Shao[2#], Xiaoqing Li[1#], Yiran Li[1], Zhi Tian[3], Fenling Zhou[1,4], Lu Chen[1,4], Jiajun Liu[1], Boyang Chang[5], Wenjian Liu[2], Hailin Tang[2]

[1]Department of Hematology, The Third Affiliated Hospital of Sun Yat-Sen University; Sun Yat-Sen Institute of Hematology; Guangzhou 510630, Guangdong Province, China;
[2]State Key Laboratory of Oncology in South China, Guangdong Provincial Clinical Research Center for Cancer, Sun Yat-Sen University Cancer Center, Guangzhou 510060, Guangdong Province, China;
[3]Taneja College of Pharmacy, University of South Florida, Tampa, FL 33620, USA;
[4]Institute of Hematology, Jinan University, Guangzhou 510632, Guangdong Province, China;
[5]Department of Interventional Radiology, The Third Affiliated Hospital of Sun Yat-Sen University, Guangzhou 510630, Guangdong Province, China

[#]These authors contributed equally to this work.
*Address for Correspondence: Hailin Tang and Wenjian Liu, State Key Laboratory of Oncology in South China, Guangdong Provincial Clinical Research Center for Cancer, Sun Yat-Sen University Cancer Center, Guangzhou 510060, Guangdong Province, China. Email: tanghl@sysucc.org.cn (H. Tang), https://orcid.org/0000-0002-3206-782X; liuwj@sysucc.org.cn (W. Liu),

Boyang Chang, Department of Interventional Radiology, The Third Affiliated Hospital of Sun Yat-Sen University, Guangzhou 510630, Guangdong Province, China. Email: changby3@mail.sysu.edu.cn

Supplementary information

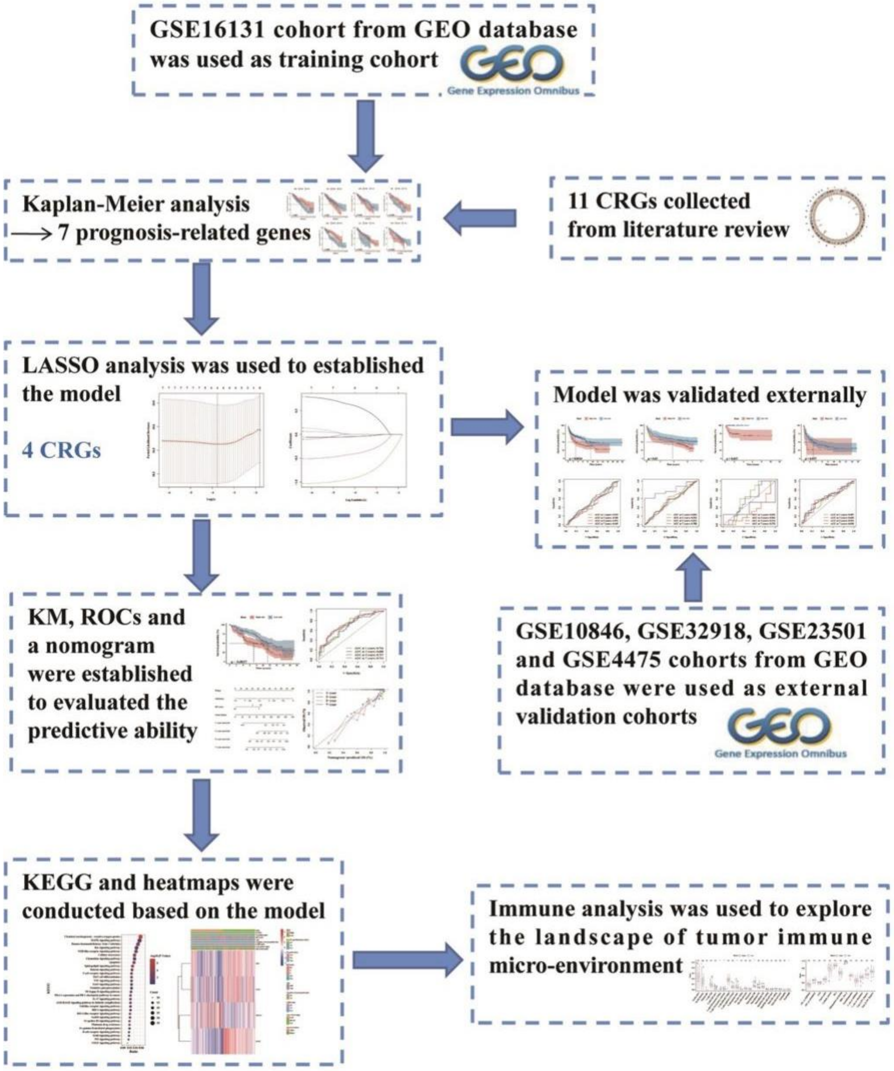

Supplementary Figure S1: Flow chart of data collection and analysis

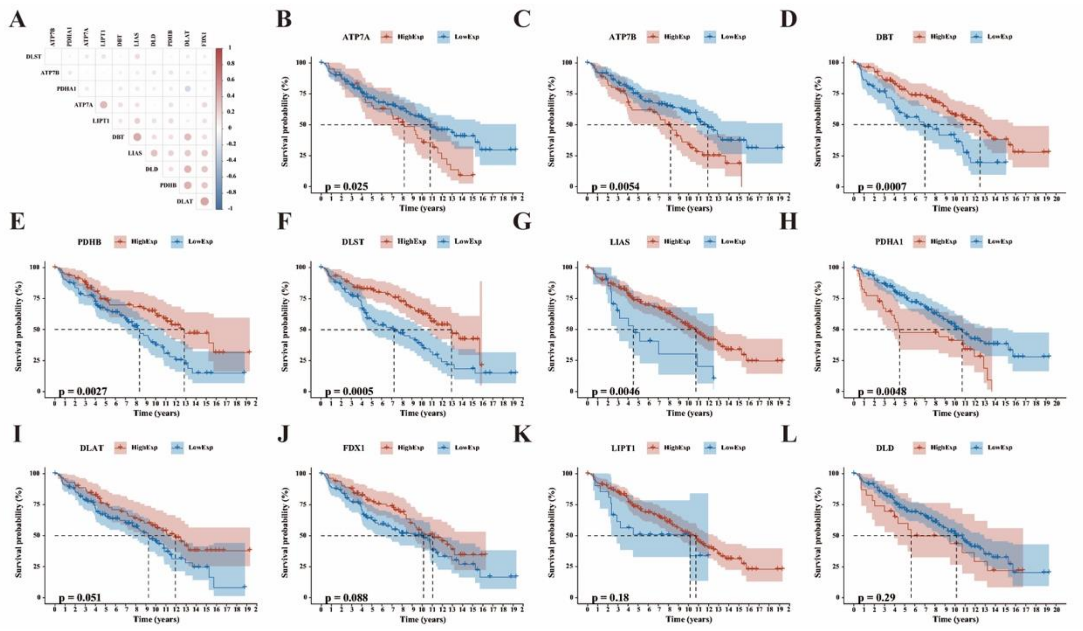

Supplementary Figure S2: Identification of prognostic CRGs in the GSE16131 cohort. **(A)** The correlation matrix plot displaying the correlation features among 11 CRGs in GSE16131 cohort. **(B-L)** Kaplan-Meier analyses of overall survival (OS) based on expression levels of 11 CRGs in GSE16131 cohort.

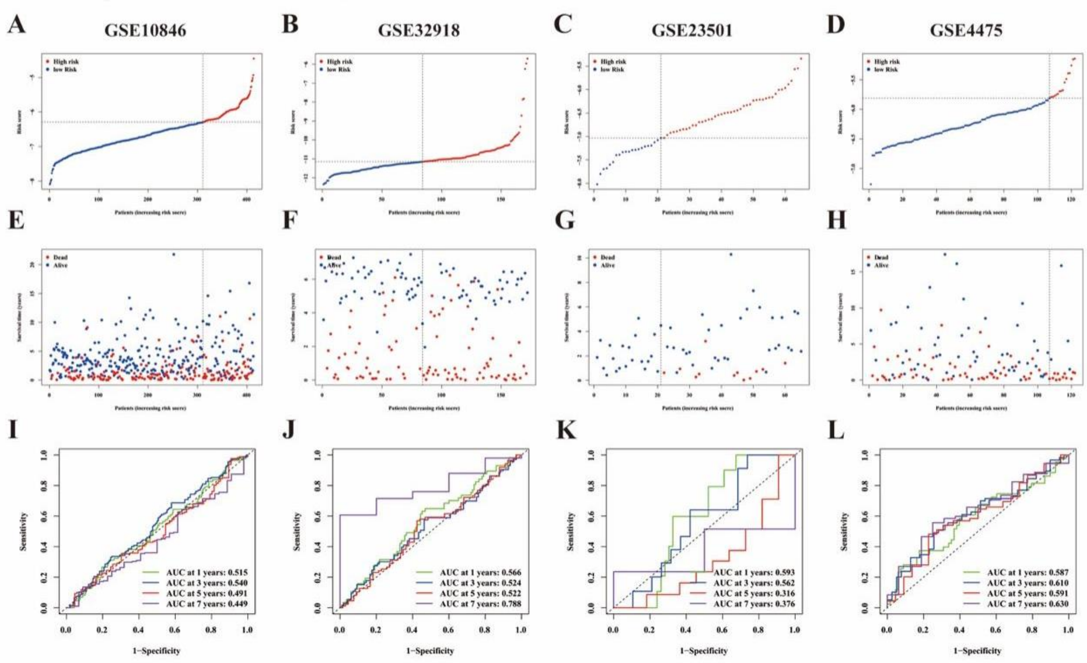

Supplementary Figure S3: Evaluation and validation of the utility of the prognostic CRG model in external validation cohorts. **(A-D)** Risk score analysis of 4-CRG

profile in the GSE10846, GSE32918, GSE23501 and GSE4475 cohorts. **(E-H)** Survival outcome analysis of 4-CRG profile in the GSE10846, GSE32918, GSE23501 and GSE4475 cohorts. **(I-L)** Time-dependent ROC analysis for 1-, 3-, 5-, and 7-year OS of 4-CRG profile in the GSE10846, GSE32918, GSE23501 and GSE4475 cohorts.
